# *Tmx2* Maintains Mitochondrial Function to Support Preimplantation Embryogenesis

**DOI:** 10.1096/fj.202500640R

**Published:** 2025-06-11

**Authors:** Shangrong Zhang, Qing Liu, Siyu Wang, Xiaoyu Zhang, Dingmei Qin, Ruisong Bai, Ji Chen, Zijian Ma, Zhipeng Lin, Yuheng Bi, Huan Liu, Aoxue Sun, Zhongzhi Mo, Hongcheng Wang, Xiaoqing Wu, Yong Liu

**Affiliations:** ^1^ Anhui Province Key Laboratory of Embryo Development and Reproductive Regulation Fuyang Normal University Fuyang Anhui China

**Keywords:** autophagy, embryo development, mice, mitochondrial dysfunction, *Tmx2*

## Abstract

Thioredoxin (TRX)‐related transmembrane proteins (TMX), a subgroup of the protein disulfide isomerase (PDI) family, comprise a class of transmembrane proteins with diverse biological functions. Among these, TMX2 (PDIA12) remains one of the least characterized members. Recent studies have identified missense mutations in TMX2 associated with aberrant brain development and cerebellar malformations, highlighting its potential importance in developmental processes. Notably, *Tmx2* mutant embryos exhibit developmental arrest at the E3.5 stage, suggesting a critical role in preimplantation embryogenesis. However, the precise molecular and cellular functions of *Tmx2* in mammalian embryonic development remain largely unexplored. In this study, we provide novel insights into the essential role of *Tmx2* during preimplantation embryonic development in mice. We demonstrate that TMX2 is specifically expressed in mouse embryos, with its subcellular localization closely associated with mitochondria during the two‐cell to eight‐cell stages. Knockdown of *Tmx2* recapitulates the phenotypic defects observed in genetic mutants, revealing a pronounced impairment in blastomere proliferation, as confirmed by EdU incorporation assays. Furthermore, TUNEL assays indicate a significant increase in apoptotic signaling in *Tmx2*‐deficient embryos, accompanied by elevated mRNA levels of the cell cycle inhibitors *p21* and *p53*. Mechanistically, we show that *Tmx2* knockdown disrupts mitochondrial function, leading to oxidative stress and impaired mitophagy and autophagy in developing embryos. These findings suggest that *Tmx2* plays a pivotal role in maintaining mitochondrial integrity and cellular homeostasis during preimplantation embryogenesis. In summary, our study elucidates the critical role of *Tmx2* in preimplantation embryonic development in mice, primarily through its regulation of mitochondrial function. These results advance our understanding of the molecular mechanisms governing preimplantation embryonic development and establish *Tmx2* as a key regulator of mitochondrial dynamics and cellular survival during this critical developmental window.

## Graphical abstract

TMX2 is a member of the thioredoxin‐related transmembrane protein family. It exhibits specific expression in mouse preimplantation embryos, and its subcellular localization is closely associated with mitochondria. Knockdown of *Tmx2* in embryos results in a reduction in mitochondrial number, a decline in mitochondrial membrane potential, and an elevation in reactive oxygen species (ROS) levels. These changes ultimately cause developmental defects in embryos by suppressing mitophagy and autophagy, thereby impairing cell proliferation and increasing apoptosis.

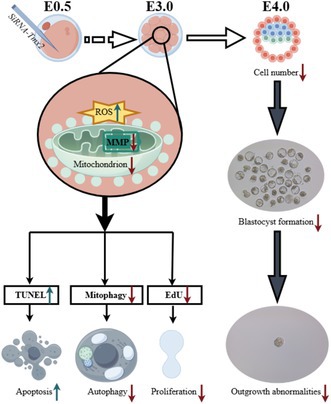

## 1 Introduction

Infertility affects approximately 10% of women of reproductive age, with early embryonic developmental arrest being a leading cause of both female infertility and recurrent miscarriage, as well as a major contributor to the failure of assisted reproductive technologies (ART) [1, 2]. Developmental arrest during the early embryonic stages often arises from disruptions in the expression of key genes critical for oogenesis or embryogenesis, which can compromise essential cellular processes and result in cleavage arrest or widespread cell death [3]. Unraveling the genetic factors and molecular mechanisms that regulate these early developmental checkpoints is therefore crucial for enhancing embryo quality and improving ART outcomes. Several factors have been implicated in developmental arrest, including genetic mutations, chromosomal abnormalities, mitochondrial dysfunction, aberrant epigenetic modifications, and various etiologies of infertility [3, 4, 5]. Among these, mitochondrial dysfunction has emerged as a central player in female reproductive health, influencing oocyte quality, fertilization, and subsequent embryonic development. Dysfunctional mitochondria can disrupt cellular energy homeostasis, trigger oxidative stress, and ultimately lead to developmental arrest and apoptosis [5, 6, 7, 8, 9, 10]. Consequently, the maintenance of functional, maternally inherited mitochondria is vital for ensuring successful early embryonic development.

Members of the protein disulfide isomerase (PDI) family, including thioredoxin‐related transmembrane proteins (TMXs), play critical roles in protein folding, unfolding, and the degradation of misfolded proteins within the endoplasmic reticulum (ER) [11, 12]. Beyond their ER‐related functions, these proteins are also essential for maintaining mitochondrial bioenergetics and cellular homeostasis. Among the PDI family members, TMX2 (also known as PDIA12) remains one of the least characterized. Unlike other PDIs, TMX2 is distinguished by a single cysteine residue in its conserved SXXC motif, which renders it devoid of oxidoreductase activity [13, 14]. TMX2 is ubiquitously expressed across various tissues, with particularly high expression levels observed in the brain, heart, liver, kidney, and pancreas [14]. It localizes to distinct subcompartments of the ER, including the nuclear envelope [15] and the mitochondria‐associated membrane (MAM) [16], where it is thought to regulate mitochondrial function. Emerging evidence underscores the importance of TMX2 in mitochondrial physiology. For instance, TMX2‐deficient fibroblasts exhibit reduced mitochondrial reserve capacity and an impaired ability to respond to oxidative stress [17]. Additionally, TMX2 has been implicated in tumor biology, where it promotes cell survival and contributes to chemotherapy resistance in hepatocellular carcinoma [18]. Recent studies have also linked TMX2 mutations to neurodevelopmental disorders, including microcephaly and other brain malformations [19, 20]. The embryonic lethality observed in homozygous *Tmx2* ^−/−^ knockout mice (C57BL/6NJ strain, Mouse Genome Informatics MGI: 1914208) further suggests that TMX2 plays a vital role in preimplantation embryonic development. However, the precise spatiotemporal expression pattern of *Tmx2* during embryogenesis, as well as the molecular mechanisms underlying its essential functions, remain poorly understood.

In this study, we employed a knockdown (KD) strategy to investigate the role of *Tmx2* in mouse embryonic development. Our findings reveal that *Tmx2* is essential for preimplantation embryogenesis and successful implantation, primarily through its role in maintaining mitochondrial function. These results offer novel insights into the molecular mechanisms by which *Tmx2* governs preimplantation embryonic development.

## 2 Materials and Methods

### 2.1 Animals

All animal care and experimental procedures conducted in this study were performed in accordance with the guidelines established by the Ethics Committee of Fuyang Normal University (Anhui, China). The mice used in this study were housed and handled in compliance with the protocols approved by the Experimental Animal Center of Fuyang Normal University. B6D2F1 mice were obtained by crossing male DBA/2 mice with female C57BL/6N mice. Both the male DBA/2 and female C57BL/6N mice were purchased from Vital River Laboratory Animal Technology Co. Ltd. in Beijing.

### 2.2 Collection of Mouse Oocytes and Preimplantation Embryos

Germinal vesicle (GV)‐stage oocytes were obtained from 8 to 12‐week‐old B6D2F1 female mice through intraperitoneal injection of 8 IU pregnant mare serum gonadotropin (PMSG; NSHF), with ovaries dissected 40 h post‐injection and follicles mechanically punctured in M2 medium (Sigma, M7167) followed by cumulus cell removal via pipetting. For MII oocyte retrieval, female mice were administered 8 IU PMSG followed by 8 IU human chorionic gonadotropin (hCG; NSHF) 47 h later. Oviducts were dissected 13 h post‐hCG administration, and the ampullary region was mechanically punctured to release cumulus‐oocyte complexes (COCs). Subsequent enzymatic digestion with 0.1 mg/mL hyaluronidase (Sigma, H4272) in M2 medium effectively dispersed cumulus cells.

To prepare zygotes for in vitro culture, siRNA microinjection, and immunofluorescence analysis, female mice were sequentially administered 8 IU PMSG and 8 IU hCG. After hCG injection, the females were paired with B6D2F1 male mice for mating. Approximately 20 h post‐hCG administration, the females were euthanized, and zygotes were collected from the ampulla of the oviduct. Cumulus cells surrounding the zygotes were removed by gentle pipetting in M2 medium supplemented with hyaluronidase. The zygotes were thoroughly washed in M2 medium and subsequently transferred to KSOM medium (prepared in the laboratory as described in Table S1) for culture. The culture medium was overlaid with sterile liquid mineral oil and maintained at 37°C in a humidified incubator with 5% CO_2_. Preimplantation embryos were collected at specified stages according to the following time points: E1.0 (2‐cell embryo), E2.0 (4‐cell embryo), E2.5 (8‐cell embryo), E3.0 (morula), and E4.0 (blastocyst), with morphological verification using differential interference contrast microscopy to ensure developmental synchronization.

### 2.3 RNA Extraction and Reverse Transcription–qPCR (RT–qPCR)

Total RNA was extracted using the Cell Amp Direct Prep Kit for RT–PCR (Real‐Time) and protein analysis reagent (Takara, 3733Q). RT‐qPCR was performed using the CFX Connect Real‐Time System (Bio‐Rad Laboratories) with the One‐Step TB Green PrimeScript PLUS RT–PCR Kit (Perfect Real Time) (Takara, RR096A). Specific primer sequences for detecting target mRNA levels are provided in Table S2. Gene expression levels were normalized to the internal control gene *H2afz* to ensure accurate quantification and comparability of results.

### 2.4 Immunofluorescence

Immunofluorescence staining was performed as previously described [20]. Briefly, embryos were fixed in 4% paraformaldehyde (PFA, Sangon Biotech, E672002), permeabilized with 0.5% Triton X‐100 (Sigma, X‐100) in PBS (Sigma, P5493/1 L) (PBST) for 30 min at room temperature, and subsequently blocked in blocking solution (PBST containing 10% fetal bovine serum (FBS, Gibco, 30 044 333)) and 1% bovine serum albumin (BSA, Sigma, B2064‐50G) for 1 h to minimize nonspecific binding. The embryos were then incubated overnight at 4°C with primary antibodies diluted in blocking solution. The primary antibodies used included: rabbit anti‐TMX2 (Origene, TA341391, 1:50), goat anti‐OCT4 (Abcam, ab27985, 1:200), mouse anti‐CDX2 (Biogenex, MU392A‐UC, 1:100), goat anti‐SOX17 (R&D Systems, AF1924, 1:100), rabbit anti‐NANOG (Abcam, ab80892, 1:50), rabbit anti‐PINK1 (Affinity, DF7742, 1:50), rabbit anti‐PARKIN (Abcepta, AP6402B, 1:50), rabbit anti‐MAP1LC3B (CUSABIO, CSB‐PA013403GA01HU, 1:50), and rabbit anti‐LAMP1 (Beyotime, AF7353, 1:100). Following primary antibody incubation, embryos were washed and incubated with a suitable secondary antibody (donkey anti‐goat IgG (H + L) highly cross‐adsorbed secondary antibody, Alexa Fluor Plus 488, Thermo Fisher, A32814; Alexa Fluor 546‐conjugated donkey anti‐rabbit IgG, Thermo Fisher, A10040; donkey anti‐mouse IgG (H + L) highly cross‐adsorbing secondary antibody, Alexa Fluor 647, Thermo Fisher, A31571) for 1 h at 37°C. Nuclei were counterstained with DAPI (Beyotime, P0131) for 10 min. Embryos were mounted and imaged using a TCS SP5 II confocal microscope (Leica, Germany). Image analysis was performed using ImageJ software (v.1.53). Negative controls, in which primary antibodies were omitted, were included to confirm the specificity of the immunofluorescence staining.

### 2.5 Mito‐Tracker Deep Red FM Fluorescence Staining

The Mito‐Tracker Deep Red FM probe (Beyotime, C1998S) is utilized to visualize mitochondrial localization, quantify mitochondrial content, and assess changes in mitochondrial membrane potential. Following the manufacturer's protocol, the Mito‐Tracker Deep Red FM dye was first diluted to a final concentration of 500 nM using KSOM solution. Fifty microliters (50 μL) droplets of KSOM medium were dispensed into a 35 mm culture dish (Falcon 351 008). The droplets were immediately overlaid with mineral oil (Sigma, M8410), followed by pre‐equilibration at 37°C in a 5% CO_2_ incubator for 2 h. After equilibration, the embryos were placed into the culture dish and incubated at 37°C for 20 min. Post‐incubation, the embryos were thoroughly rinsed five times with M2 medium. To visualize the embryonic nuclei, the samples were stained with an anti‐fade mounting medium containing DAPI and mounted on glass slides for imaging using a Leica TCS SP5 II confocal microscope. In some cases, the embryos were fixed with 4% PFA for subsequent co‐staining with additional fluorescent dyes.

### 2.6 Microinjection

Microinjection was carried out following established protocols [21]. Briefly, the procedure was performed using an inverted microscope (Nikon, Japan) equipped with a piezo‐driven micromanipulator (Prime Tech, Japan). A volume of 5–10 picoliters (pL) of either 80 μM scrambled control siRNA or *Tmx2* siRNA was microinjected into the target cells. The scrambled control siRNA sequences were as follows: sense, 5′‐UUCUCCGAACGUGUCACGUTT‐3′, and antisense, 5′‐ACGUGACACGUUCGGAGAATT‐3′. For *Tmx2* siRNA, three distinct sequences were used: siRNA1: sense, 5′‐GCUCUGCUUUCCAUUGCUUTT‐3′, and antisense, 5′‐AAGCAAUGGAAAGCAGAGCTT‐3′ 3′; siRNA2: sense, 5′‐GCCAAUCCUUUGCUCCCAUTT‐3′, and antisense, 5′‐AUGGGAGCAAAGGAUUGGCTT‐3′; siRNA3: sense, 5′‐GGCCGCAGAUUGACAAGAATT‐3′, and antisense, 5′‐GGCCGCAGAUUGACAAGAATT‐3′. All siRNA sequences were synthesized by Genepharma. The microinjection setup and parameters were optimized to ensure precise delivery of siRNA into the target embryos.

### 2.7 Outgrowth Assay

Blastocysts were individually harvested and cultured in separate wells containing DMEM (Gibco, 11 960–051) supplemented with 10% fetal bovine serum and 100X GlutaMAX (Thermo, 35 050 061). The blastocysts were incubated in a humidified environment at 37°C with 5% CO_2_ for a period of 3 days to facilitate attachment and subsequent outgrowth, after which imaging was conducted. The assessment of outgrowths was based on morphological criteria. Specifically, a normal outgrowth was characterized by the presence of a distinct inner cell mass (ICM) colony encircled by a trophoblast monolayer. Conversely, embryos that failed to hatch or exhibited outgrowths devoid of either an ICM colony or a trophoblast monolayer were classified as failed.

### 2.8 EdU Incorporation Assay

EdU incorporation was performed using the Cell‐Light EdU Apollo567 In Vitro Kit (RiboBio, C10310‐1), followed by DAPI staining. Live embryos were incubated in KSOM medium containing 1 mM EdU (5‐ethynyl‐2′‐deoxyuridine) for 2 h. After incubation, the embryos were fixed with 4% paraformaldehyde for 30 min and then permeabilized using 0.5% Triton X‐100 in PBS at room temperature for 30 min. Following permeabilization, the embryos were treated with a freshly prepared 1× Apollo reaction cocktail and incubated at room temperature for 30 min in the dark. Subsequently, the embryos were counterstained with DAPI to visualize nuclei. Imaging was performed using a Leica TCS SP5 II confocal microscope, with an excitation wavelength of 550 nm and an emission wavelength of 565 nm for EdU detection.

### 2.9 TUNEL Assay

Embryo apoptosis was evaluated using a terminal deoxynucleotidyl transferase‐mediated dUTP nick‐end labeling (TUNEL) assay kit (Beyotime Biotechnology, C1086). Briefly, embryos were fixed in 4% paraformaldehyde at room temperature for 60 min and then permeabilized with 0.5% Triton X‐100 in PBS for 30 min. After permeabilization, the embryos were washed three times in PBST. They were subsequently incubated in a reaction mixture containing 10% terminal deoxynucleotidyl transferase and 90% fluorescein‐dUTP at 37°C for 1 h in the dark. Following five additional washes in PBST, the embryos were counterstained with 10 μg/mL Hoechst 33342 for 10 min in the dark to label nuclei. Apoptotic cells were visualized using a Leica TCS SP5 II confocal microscope, with excitation wavelengths of 488 nm for TUNEL and 405 nm for Hoechst 33342.

### 2.10 Mitochondrial Membrane Potential Assay

The embryos were incubated in KSOM medium supplemented with 20 μg/mL JC‐1 (5,5′,6,6′‐tetrachloro‐1,1′,3,3′‐tetraethylbenzimidazolylcarbocyanine iodide; Beyotime, C2005) for 30 min at 37°C under 5% CO_2_, following the manufacturer's protocol. After incubation, the embryos were washed twice in KSOM and transferred to a glass‐bottomed cell culture dish containing a drop of HEPES‐CZB medium. Imaging was performed using a Leica TCS SP5 II confocal microscope. Mitochondrial membrane potential was assessed by calculating the ratio of red fluorescence intensity (representing activated mitochondria, J‐aggregates) to green fluorescence intensity (representing less‐activated mitochondria, J‐monomers). The fluorescence intensity was quantified using ImageJ software (v.1.53).

### 2.11 ROS Measurement

To visualize reactive oxygen species (ROS) levels, embryos were incubated in KSOM medium supplemented with 5 μM DCFH‐DA (Beyotime, S0033S) for 30 min at 37°C under 5% CO_2_, following the manufacturer's instructions. After incubation, the embryos were washed three times in 0.1% PVA‐PBS and mounted on glass slides. Fluorescence imaging was performed using a Leica TCS SP5 II confocal microscope, with an excitation wavelength of 488 nm and an emission wavelength of 525 nm. The fluorescence intensity, reflecting ROS levels, was quantified using ImageJ software (v.1.53).

### 2.12 Statistical Analysis

All experiments were repeated at least three times with independent embryo collections, and a minimum of 10 oocytes or embryos per group were used for each experiment. Statistical analyses were performed using GraphPad Prism 8.3.0, with one‐way ANOVA followed by Dunnett's test for multiple comparisons. Data are presented as mean ± SEM, and a *p* < 0.05 was considered statistically significant.

## 3 Results

### 3.1 Expression and Localization of *Tmx2* in Mouse Oocytes and Preimplantation Embryos

**FIGURE 1:**
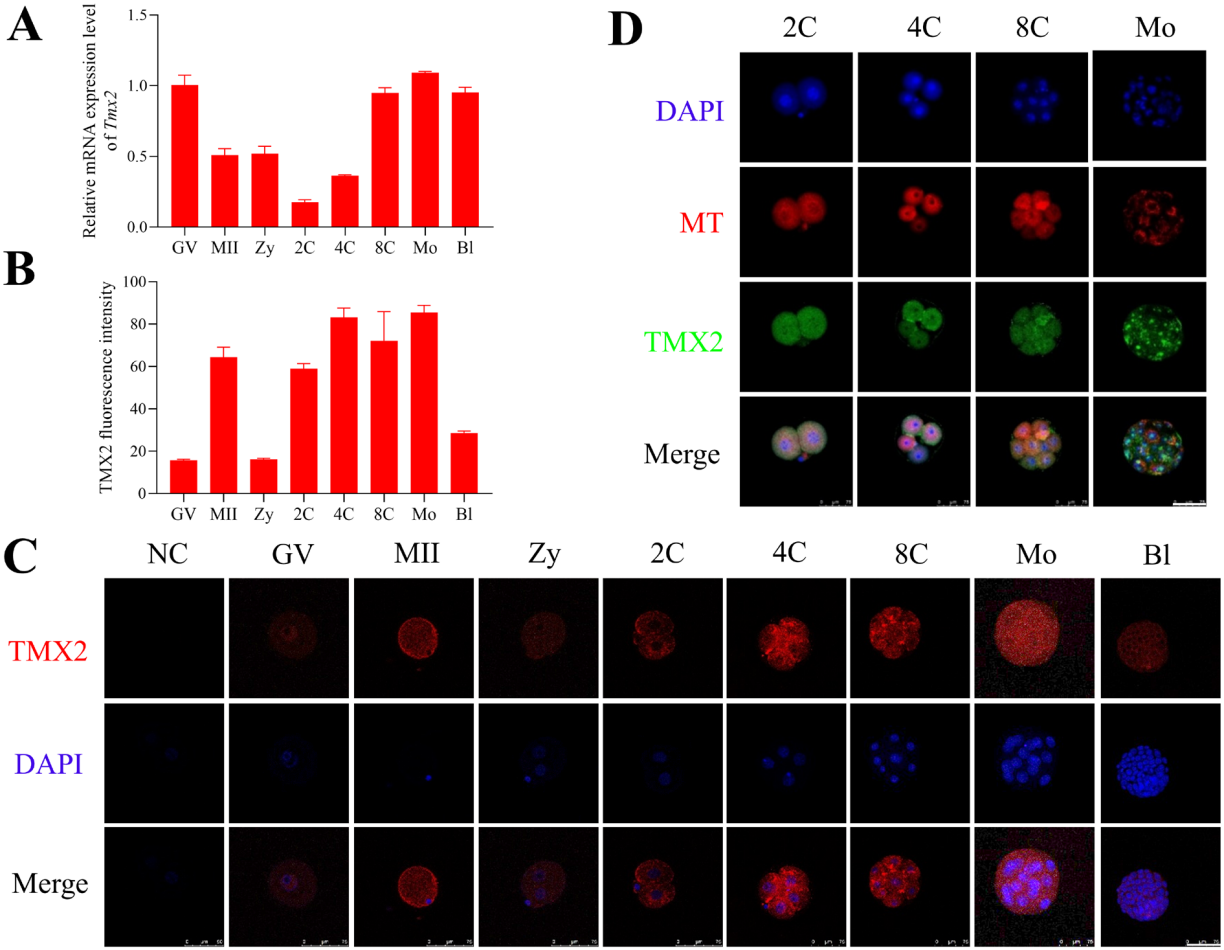
Expression and localization of *Tmx2* in mouse oocytes and preimplantation embryos. (A) RT–qPCR analysis of *Tmx2* mRNA expression from germinal vesicle (GV)‐stage oocytes to the blastocyst stage. 2C, 2‐cell embryo; 4C, 4‐cell embryo; 8C, 8‐cell embryo; Bl, Blastocyst; GV, Germinal vesicle‐stage oocyte; MII, Metaphase II oocyte; Mo, Morula; Zy, Zygote. The relative expression level of *Tmx2* is presented as fold change normalized to *H2afz*. (B) Quantification of TMX2 fluorescence intensity using ImageJ. The number of samples analyzed at each stage was as follows: GV = 11, MII = 10, Zy = 20, 2C = 13, 4C = 20, 8C = 12, Mo = 23, and Bl = 12. (C) Immunofluorescence staining of TMX2 (red) and nuclear DNA (blue) from GV‐stage oocytes to the blastocyst stage. NC, The negative control (no primary antibody); Scale bars: 75 μm. (D) Co‐localization of TMX2 (immunostained with anti‐TMX2 antibody) and mitochondria (stained with MitoTracker Deep Red FM) in embryos. Scale bar: 75 μm.

We initially utilized RT‐qPCR to examine the expression dynamics of *Tmx2* during preimplantation development. The results indicated that *Tmx2* transcription levels decreased immediately after fertilization but were subsequently upregulated starting from the 4‐cell stage, peaking at the morula stage (Figure 1A). To further investigate the expression and subcellular localization of TMX2 protein during preimplantation development, immunofluorescence staining was performed. The findings revealed that TMX2 protein was highly expressed at the 4‐cell, 8‐cell, and morula stages, with predominant localization in the cytoplasm, external to the nucleus (Figure 1B,C). To determine whether TMX2 colocalized with mitochondria, embryos were stained with the mitochondrial fluorescent probe MitoTracker Deep Red FM. The results demonstrated that TMX2 colocalized with mitochondria at the 2‐cell, 4‐cell, and 8‐cell stages. However, during the morula stage, TMX2 exhibited partial colocalization with mitochondria, along with minor ectopic expression (Figure 1D).

### 3.2 *Tmx2* Knockdown Affects Mouse Preimplantation Embryo Development

**FIGURE 2:**
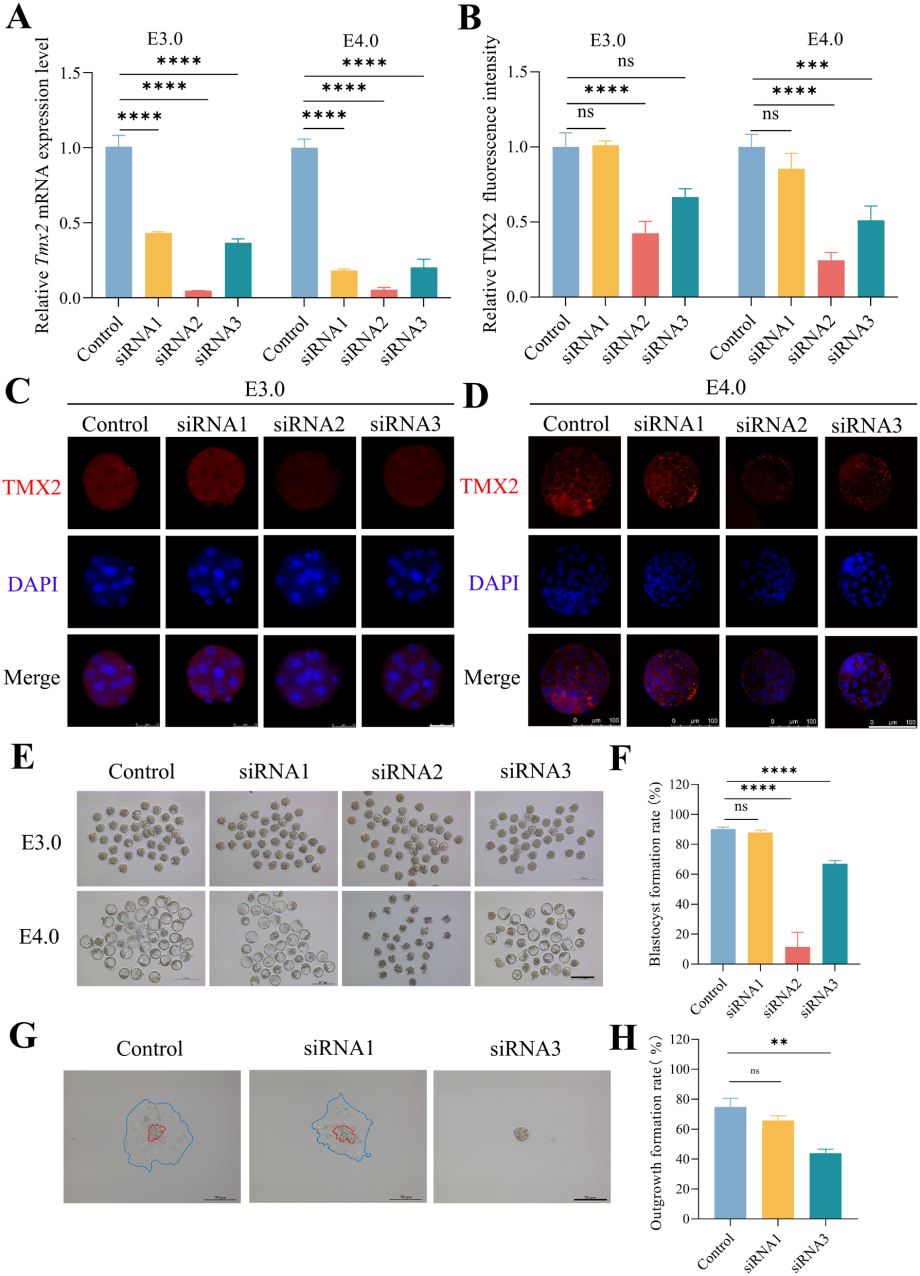
Effects of *Tmx2* knockdown on embryonic development. (A) RT‐qPCR analysis confirmed *Tmx2* knockdown efficiency in E3.0 (morula) and E4.0 (blastocyst) embryos, with *H2afz* serving as the internal control (B) Quantification of relative TMX2 fluorescence intensity. The number of samples analyzed at each stage was as follows: At E3.0:Control = 11, siRNA1 = 12, siRNA2 = 10, siRNA3 = 11; at E4.0: Control = 18, siRNA1 = 13, siRNA2 = 16, siRNA3 = 10. (C) Representative immunofluorescence images depicting TMX2 expression at E3.0embryos following *Tmx2* knockdown. Scale bar: 75 μm. (D) Representative immunofluorescence images depicting TMX2 expression at E4.0 embryos following *Tmx2* knockdown. Scale bar: 100 μm. (E) Morphological comparison of E3.0 and E4.0 embryos between the control and *Tmx2*‐knockdown groups. Scale bars: 200 μm. (F) Blastocyst formation rates in the control and *Tmx2*‐knockdown groups. The number of samples analyzed in each group was as follows: Control = 197, siRNA1 = 172, siRNA2 = 203, siRNA3 = 210. (G) Outgrowth cultures derived from *Tmx2*‐knockdown embryos. Scale bars: 200 μm. (H) Outgrowth formation rates in the control (*n* = 117) and *Tmx2*‐knockdown (siRNA1 = 78, siRNA3 = 72) groups. Statistical significance is indicated as follows: **p < 0.01, ***p < 0.001 and ****p < 0.0001.

In this study, a *Tmx2* knockdown embryo model was established using microinjection and RNA interference (RNAi) technology. The knockdown efficiency of *Tmx2* siRNAs was validated through RT‐qPCR and immunofluorescence staining. As shown in Figure 2A, the quantitative results demonstrated that *Tmx2* transcriptional levels in siRNA1‐, siRNA2‐, and siRNA3‐treated groups were significantly reduced compared to the control group at both embryonic stages E3.0 and E4.0. Immunofluorescence quantification across embryonic developmental stages revealed distinct TMX2 protein knockdown profiles among the siRNA groups (Figure 2B–D). While siRNA1 showed no statistically significant reduction in TMX2 protein levels at either E3.0 or E4.0, siRNA2 demonstrated robust and consistent suppression, achieving 57.5% (*p* < 0.0001) and 75.4% (*p* < 0.0001) knockdown efficiencies at E3.0 and E4.0, respectively. Notably, siRNA3 exhibited stage‐dependent efficacy, with minimal protein reduction at E3.0 (33.3%, *p* = 0.168) that significantly improved to 49.7% (*p* < 0.001) at E4.0.

Morphological analysis demonstrated stage‐specific developmental consequences of *Tmx2* knockdown: siRNA1‐treated embryos showed comparable blastocyst formation rates to controls, whereas siRNA2 caused near‐complete developmental arrest, with siRNA3 exhibiting intermediate impairment (Figure 2E,F). Subsequent in vitro outgrowth assays revealed functional parallels‐siRNA1 maintained developmental competence, while 50% of embryos in the siRNA3 group failed to hatch from the zona pellucida, preventing further development (Figure 2G,H). These results establish a functional hierarchy where siRNA2's superior knockdown efficiency drives complete developmental blockade, siRNA3's partial efficacy permits limited developmental progression, and siRNA1 failed to reduce *Tmx2* levels sufficiently for phenotypic effects. These findings collectively indicate that *Tmx2* knockdown significantly impairs preimplantation embryonic development and blastocyst implantation potential in mice.

### 3.3 *Tmx2* Deficiency Does Not Influence Embryonic Lineage Differentiation

**FIGURE 3:**
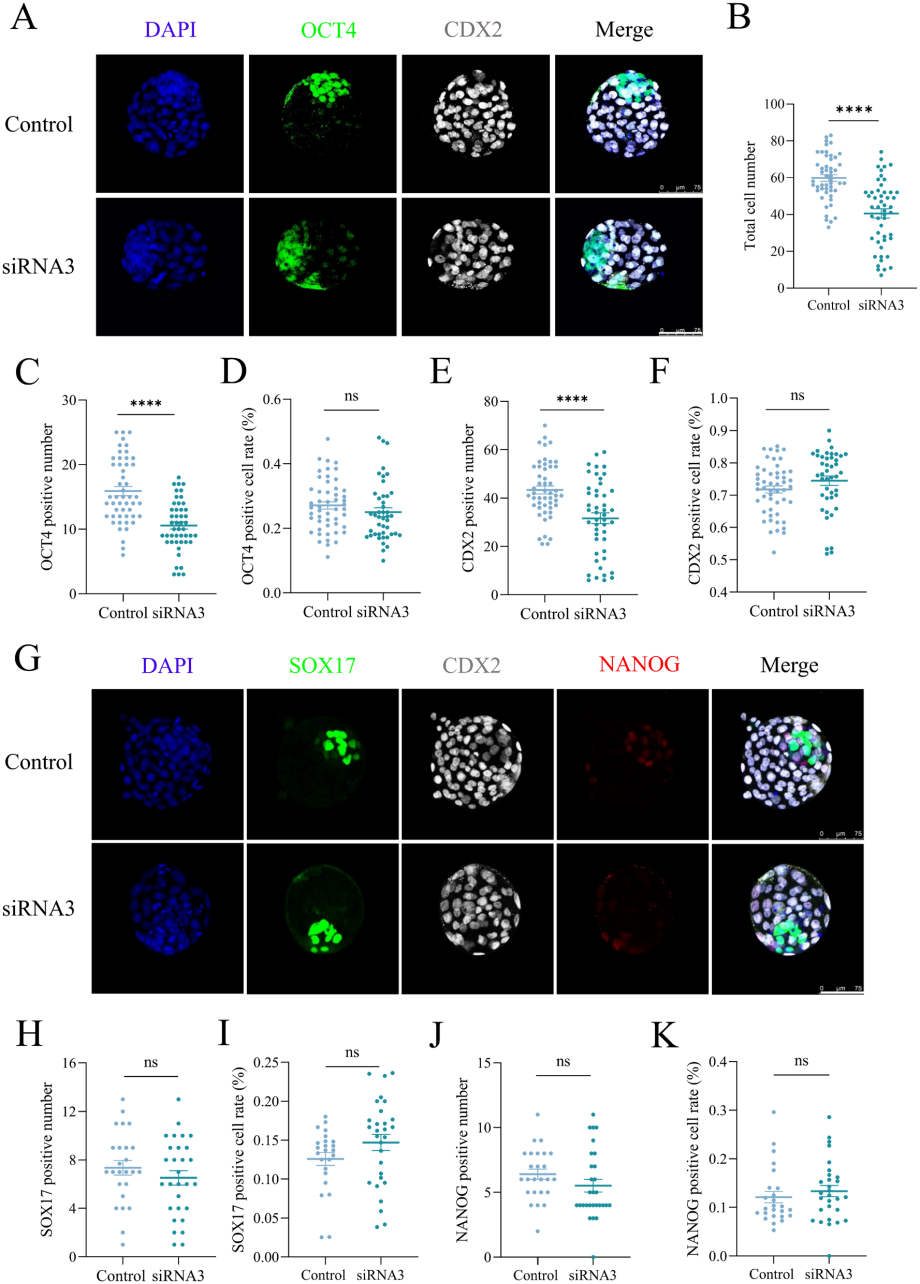
*Tmx2* knockdown did not affect ICM/TE or PE/EPI lineage specification. (A) Immunofluorescence (IF) staining of OCT4 (ICM marker) and CDX2 (TE marker) in control (*n* = 50) and *Tmx2*‐siRNA3 knockdown (*n* = 47) blastocysts at E4.0. (B, C, E) Quantification of total cell number per blastocyst (B), OCT4‐positive cells (C), and CDX2‐positive cells (E) revealed a significant reduction in *Tmx2*‐knockdown embryos. (D, F) The percentages of OCT4‐positive (D) and CDX2‐positive (F) cells showed no significant difference between the control and *Tmx2*‐knockdown groups. (G) IF staining of SOX17 (PE marker), NANOG (EPI marker), and CDX2 (TE marker) in control (*n* = 25) and *Tmx2*‐knockdown (*n* = 29) blastocysts at E4.0. (H–K) No significant changes were observed in the number of SOX17‐positive PE cells (H), NANOG‐positive EPI cells (J), or the percentages of SOX17‐positive PE cells (I) and NANOG‐positive EPI cells (K). Statistical significance is indicated as follows: ****p < 0.0001.

To explore the mechanisms underlying embryonic lethality in vitro, an indirect immunofluorescence assay was employed to assess the expression of lineage differentiation markers, including OCT4 (ICM), CDX2 (TE), NANOG (EPI), and SOX17 (PrE), in blastocysts. Given that embryos subjected to siRNA2 knockdown failed to develop into blastocysts, siRNA3‐knockdown embryos were selected for this experiment. As shown in Figure 3A,C,E, *Tmx2* knockdown led to a reduction in the number of ICM‐ and TE‐positive cells. However, the total cell number in *Tmx2*‐knockdown embryos was also decreased (Figure 3B). To further evaluate lineage differentiation, the proportions of ICM‐ and TE‐positive cells relative to the total cell number were calculated, revealing no significant changes in these proportions (Figure 3D,F). These results suggest that *Tmx2* deletion does not impair the differentiation of ICM and TE lineages in blastocysts. Additionally, no significant differences were observed in the number or proportion of EPI‐ and PrE‐positive cells among total blastocyst cells (Figure 3G–K), indicating that *Tmx2* knockdown does not affect the differentiation of EPI and PrE lineages. Collectively, these findings demonstrate that *Tmx2* knockdown does not disrupt embryonic lineage differentiation.

### 3.4 Knockdown of *Tmx2* Impairs Cell Proliferation and Induces Apoptosis

**FIGURE 4:**
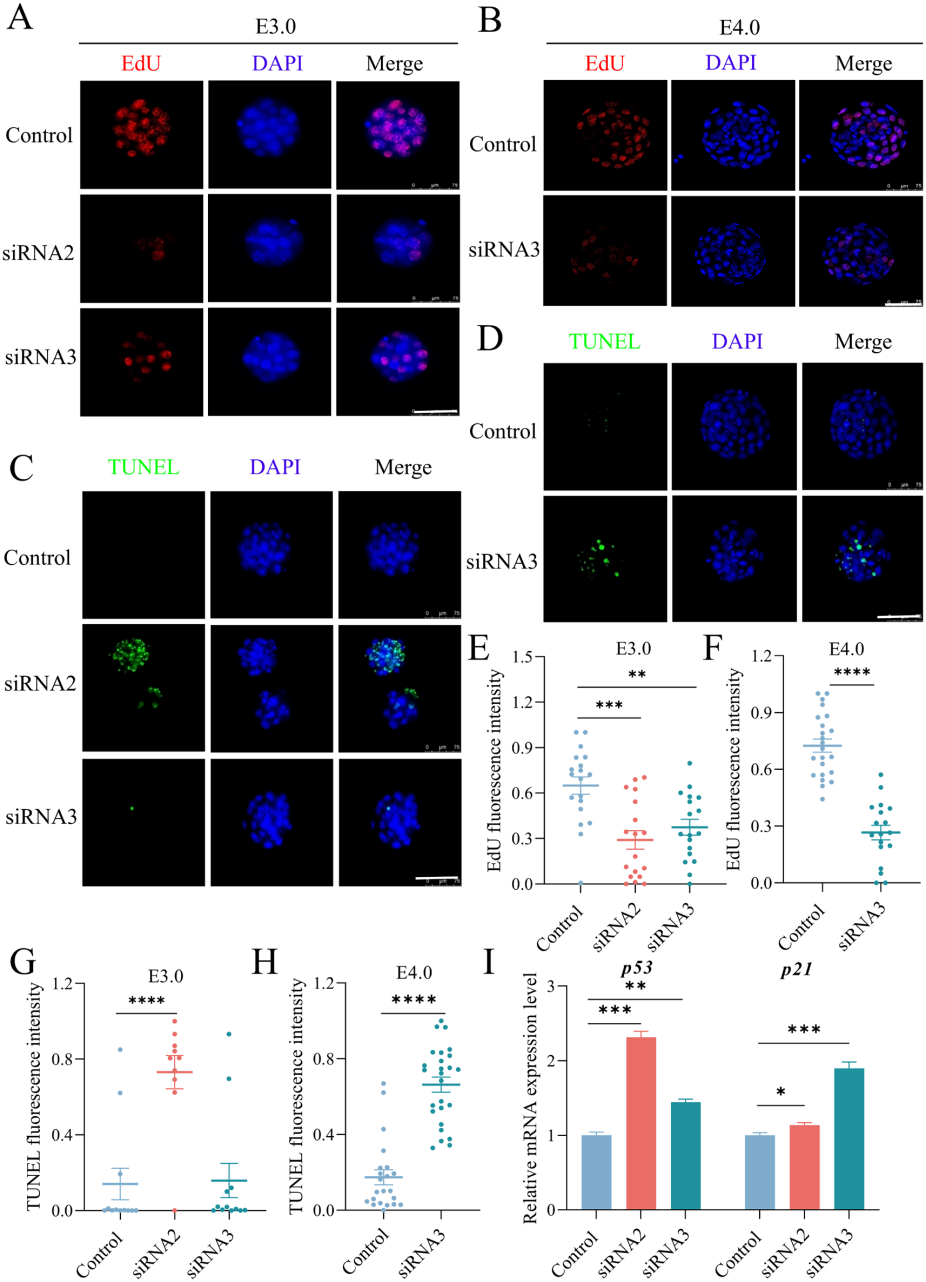
*Tmx2* knockdown inhibits cell proliferation and induces apoptosis in embryos. (A, B) Representative images of control and *Tmx2*‐knockdown embryos at E3.0 and E4.0, showing EdU‐positive signals. Scale bars: 75 μm. (C, D) TUNEL assays of control and *Tmx2*‐knockdown embryos at E3.0 and E4.0. Scale bars: 75 μm. (E) Proliferative ability index (ratio of EdU‐positive nuclei to total nuclei) in control and *Tmx2*‐knockdown groups at E3.0. Sample sizes: Control = 19, siRNA2 = 18, siRNA3 = 18. (F) Proliferative ability index in control (*n* = 23) and *Tmx2*‐siRNA3 knockdown (*n* = 18) groups at E4.0. (G) Quantification of apoptotic signals in control and *Tmx2*‐knockdown embryos at E3.0. Sample sizes: Control = 12, siRNA2 = 10, siRNA3 = 12. (H) Quantification of apoptotic signals in control (*n* = 22) and *Tmx2*‐siRNA3 knockdown (*n* = 26) embryos at E4.0. (I) RT–qPCR analysis of *p21* and *p53* mRNA levels at E3.0 in control and *Tmx2*‐knockdown embryos. Statistical significance: *p < 0.05, **p < 0.01, ***p < 0.001, and ****p < 0.0001.

The total cell count results (Figure 3B) suggest that *Tmx2* knockdown may affect preimplantation embryonic development by modulating cell proliferation or apoptosis. To explore this possibility, we first examined the impact of *Tmx2* knockdown on blastomere proliferation using EdU incorporation assays (Figure 4A,B). A significant reduction in the proliferation rate was observed in *Tmx2* knockdown embryos starting from the E3.0 stage (Figure 4E,F). These findings demonstrate that *Tmx2* knockdown markedly inhibits embryonic cell proliferation at both the E3.0 and E4.0 stages.

Furthermore, apoptotic cells were identified using TUNEL staining (Figure 4C,D). As shown in Figure 4G,H, the proportion of TUNEL‐positive signals relative to the total number of nuclei was significantly higher in siRNA2‐knockdown embryos compared to control embryos at E3.0. At this stage, siRNA3 knockdown did not significantly affect apoptosis, likely due to its lower knockdown efficiency compared to siRNA2, resulting in a delayed impact on embryonic development. To further investigate, apoptosis was assessed in siRNA3‐knockdown embryos at E4.0, revealing that siRNA3 knockdown indeed induced apoptosis. These results collectively indicate that *Tmx2* knockdown triggers apoptosis in embryos.

P21, a well‐known cell cycle repressor, is a key downstream target of P53. Elevated *p53* transcription can lead to cell cycle arrest and apoptosis. To explore this mechanism, the expression levels of *p53* and *p21* were quantitatively analyzed. As shown in Figure 4I, both *p53* and *p21* were significantly upregulated in the *Tmx2*‐knockdown group. These findings suggest that *Tmx2* knockdown disrupts embryonic cell proliferation and induces apoptosis.

### 3.5 *Tmx2* Knockdown Disrupts Mitochondrial Function and Increases Oxidative Stress in Arrested Morula‐Stage Embryos

**FIGURE 5:**
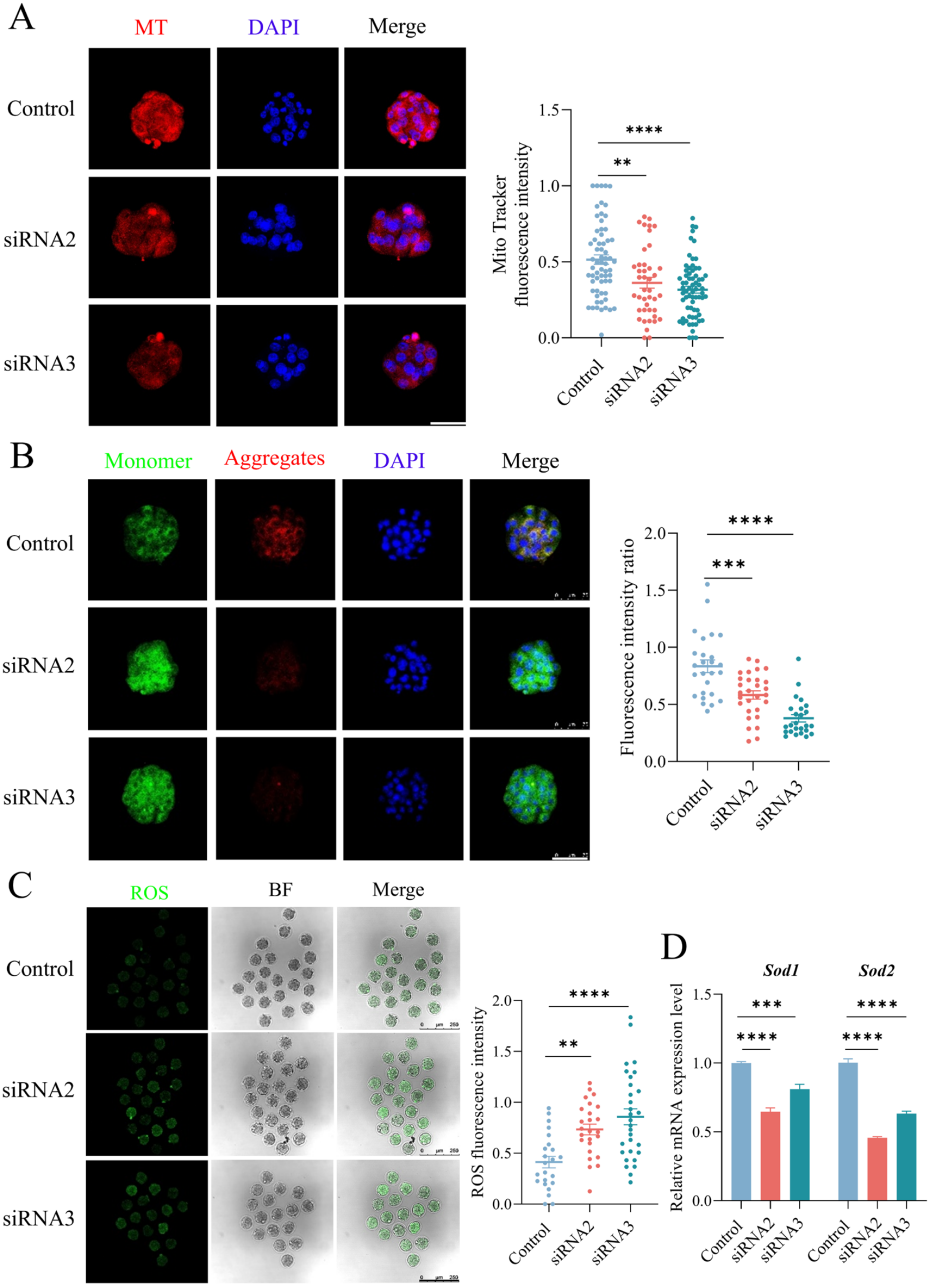
*Tmx2* knockdown disturbs mitochondrial dysfunction and increases ROS level in morula‐stage embryos. (A) Mito‐Tracker Deep Red FM staining (scale bars: 75 μm) reveals decreased mitochondrial intensity in *Tmx2*‐knockdown morula‐stage embryos compared to controls. Sample sizes: Control = 64, siRNA2 = 42, siRNA3 = 68. (B) Representative JC‐1 staining images (scale bars: 75 μm) of control and *Tmx2*‐knockdown morula‐stage embryos demonstrate a lower aggregate‐to‐monomer ratio in *Tmx2*‐knockdown embryos, reflecting impaired mitochondrial membrane potential. Sample sizes: Control = 26, siRNA2 = 28, siRNA3 = 25. (C) DCFH‐DA staining (scale bar: 200 μm) reveals elevated ROS levels in *Tmx2*‐knockdown embryos, as quantified by increased fluorescence intensity compared to controls. Sample sizes: Control = 23, siRNA2 = 25, siRNA3 = 30. (D) RT‐qPCR analysis of *Sod1* and *Sod2* mRNA expression in control and *Tmx2*‐knockdown embryos. Statistical significance: **p < 0.01, ***p < 0.001 and ****p < 0.0001.

As shown in Figure 1D, TMX2 colocalized with mitochondria from the 2‐cell to 8‐cell stages, and mitochondria are the primary endogenous sources of reactive oxygen species (ROS). Based on this, we hypothesized that *Tmx2* knockdown might impair mitochondrial function and increase oxidative stress. To evaluate mitochondrial function, we first assessed the number of mitochondria and their membrane potential. As shown in Figure 5A, a significant decrease in the fluorescence intensity of MitoTracker Deep Red FM was observed in *Tmx2*‐knockdown embryos, indicating a reduction in both mitochondrial quantity and membrane potential.

We further measured mitochondrial membrane potential using JC‐1 dye. As shown in Figure 5B, *Tmx2* knockdown increased the formation of JC‐1 monomers and decreased the number of aggregates in morula‐stage embryos. Quantitative analysis revealed that the fluorescence intensity ratio of aggregates to monomers was significantly lower in the *Tmx2*‐knockdown group compared to the control group, confirming impaired mitochondrial function in *Tmx2*‐knockdown embryos.

Aberrant mitochondrial function is often associated with elevated oxidative stress. Consistent with this, our results demonstrated that *Tmx2* knockdown led to a significant increase in intracellular ROS levels (Figure 5C). Additionally, the mRNA expression levels of the antioxidant enzymes Sod1 and Sod2 were markedly reduced in *Tmx2*‐knockdown embryos compared to controls (Figure 5D). Together, these findings indicate that *Tmx2* knockdown exacerbates mitochondrial dysfunction and induces oxidative stress in embryos, which may contribute to the observed developmental defects.

### 3.6 *Tmx2* Knockdown Inhibited Mitophagy and Autophagy in Morula‐Stage Embryos

**FIGURE 6:**
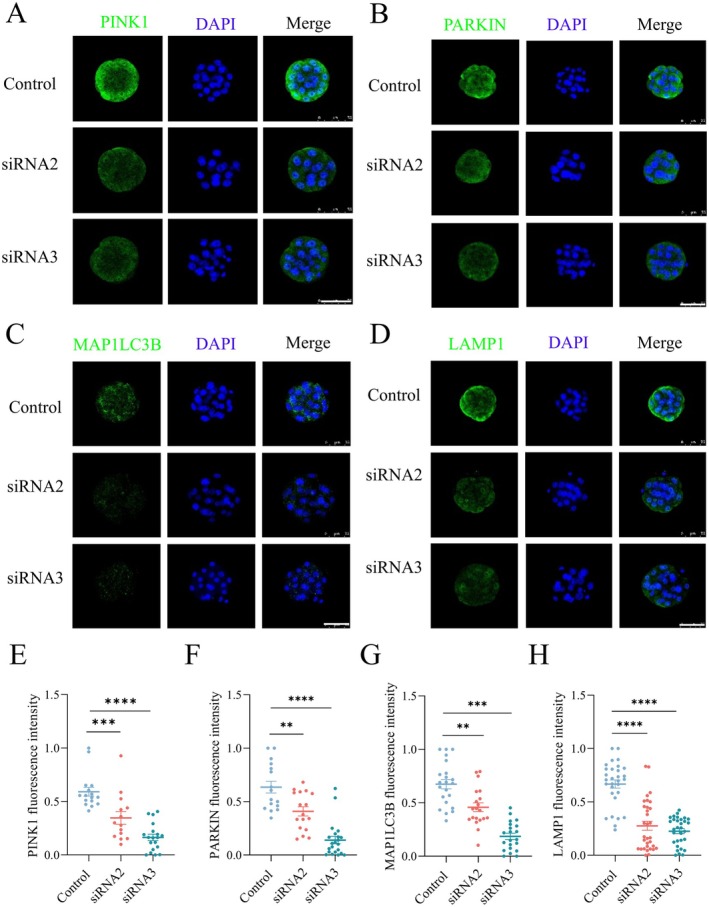
*Tmx2* knockdown inhibits mitophagy and autophagy in morula‐stage embryos. (A‐D) Immunostaining for PINK1 (A), PARKIN (B), MAP1LC3B (C), and LAMP1 (D) in control and *Tmx2*‐knockdown embryos. Scale bars: 75 μm. (E‐H) Quantification of immunofluorescence intensity for PINK1 (E), PARKIN (F), MAP1LC3B (G), and LAMP1 (H) in control and *Tmx2*‐knockdown morula‐stage embryos. Sample sizes: PINK1: Control = 16, siRNA2 = 14, siRNA3 = 19; PARKIN: Control = 21, siRNA2 = 20, siRNA3 = 23; MAP1LC3B: Control = 22, siRNA2 = 20, siRNA3 = 21; LAMP1: Control = 30, siRNA2 = 32, siRNA3 = 31. Statistical significance: **p < 0.01, ***p < 0.001 and ****p < 0.0001.

TMX2 was demonstrated to promote cytoprotective mitophagy and macroautophagy/autophagy during oxidative stress [18]. Based on this, we hypothesized that *Tmx2* knockdown could inhibit mitophagy and autophagy in preimplantation embryos. To test this hypothesis, we performed immunostaining for PINK1 and PARKIN, key mediators of mitophagy [22, 23, 24]. The results showed that the protein expression levels of PINK1 and PARKIN were significantly reduced in *Tmx2*‐knockdown embryos compared to controls (Figure 6A,B). To further assess autophagy and mitophagy inhibition, we examined the expression of MAP1LC3B, a critical marker for autophagosome formation [25]. *Tmx2*‐knockdown embryos exhibited decreased expression of MAP1LC3B, indicating a reduction in autophagosome formation (Figure 6E,G). Since autophagosomes typically fuse with lysosomes to degrade abnormal intracellular proteins or damaged organelles [26], we also evaluated lysosomal content by immunostaining for LAMP1, a lysosomal and late endosomal marker. The results revealed a significant downregulation of LAMP1‐positive signals in *Tmx2*‐knockdown embryos (Figure 6F,H), suggesting impaired lysosomal function and autophagy.

Collectively, these findings demonstrate that *Tmx2* knockdown inhibits both mitophagy and autophagy in morula‐stage embryos, highlighting the critical role of *Tmx2* in maintaining these essential cellular processes during preimplantation embryonic development.

## 4 Discussion

The importance of preimplantation embryonic development in reproductive health cannot be overstated, as anomalies during this critical phase can lead to infertility, miscarriage, or congenital defects. Preimplantation embryogenesis is a highly regulated process involving tightly controlled cellular events, including proliferation and differentiation. In this study, we elucidated the essential role of *Tmx2* in preimplantation embryonic development in mice. Knockdown of *Tmx2* through microinjection of siRNA into one‐cell zygotes significantly reduced the blastocyst formation rate and compromised hatching ability, which is indicative of impaired developmental potential. *Tmx2* knockdown disrupted mitochondrial function and induced oxidative stress, leading to reduced proliferation and, ultimately, apoptosis, resulting in embryonic arrest.

The immunofluorescence results indicated that *Tmx2* knockdown had no significant impact on embryonic lineage differentiation. However, further analysis of developmentally arrested embryos in the knockdown group showed a notable reduction in the total number of blastomeres per embryo at E3.0 and E4.0, suggesting that *Tmx2* interference may impair cell proliferation or enhance apoptosis. Previous studies have reported that TMX2 promotes hepatocellular carcinoma (HCC) growth and suppresses apoptosis [18]. In line with this, our findings revealed that *Tmx2* deficiency led to a decreased rate of blastomere cell division, as confirmed by EdU assay results. Additionally, *Tmx2* knockdown during preimplantation embryo development resulted in elevated levels of TUNEL, *p53*, and *p21*, further supporting the induction of apoptosis. Taken together, these findings demonstrate that *Tmx2* knockdown disrupts blastomere proliferation and triggers apoptosis in preimplantation embryos.

The dynamic organization of mitochondria with a high membrane potential is essential for proper embryo development [27, 28]. In mouse preimplantation embryos, TMX2 exhibits subcellular localization at the 2‐, 4‐, and 8‐cell stages, potentially associating with mitochondria‐associated endoplasmic reticulum membranes (MAMs). This observation aligns with findings in other cell types, such as A375P and HeLa cells [16], underscoring the critical role of *Tmx2* in mitochondrial regulation. Disruptions in mitochondrial function are often characterized by a decrease in mitochondrial quantity and a reduction in mitochondrial membrane potential. Consistent with this, *Tmx2*‐knockdown embryos displayed a reduction in both mitochondrial quantity and membrane potential. Dysfunctional mitochondria can produce excessive reactive oxygen species (ROS) and release apoptosis‐inducing factors, leading to cell death and developmental abnormalities [29]. Furthermore, elevated ROS levels can exacerbate mitochondrial dysfunction, creating a detrimental cycle. In mammalian embryos, excessive ROS concentrations are frequently linked to developmental arrest or in vitro damage [30]. Previous studies have highlighted the role of *Tmx2* in maintaining redox homeostasis [17]. In this study, *Tmx2* knockdown resulted in increased ROS levels and decreased mRNA expression of *Sod1* and *Sod2*, confirming the induction of oxidative stress. These findings collectively suggest that *Tmx2* knockdown impairs embryonic development by disrupting mitochondrial function and elevating ROS levels.

Mitophagy is a vital process for maintaining mitochondrial quality and regulating mitochondrial number. It involves the selective engulfment of damaged mitochondria by autophagosomes, which are subsequently degraded by lysosomes in response to the loss of mitochondrial membrane potential [31]. Studies have shown that impaired autophagy significantly reduces the rate of embryonic development and the total cell number while increasing the rate of apoptosis [32]. Furthermore, autophagy defects have been linked to the accumulation of damaged mitochondria and elevated reactive oxygen species (ROS), ultimately leading to apoptosis, as observed in NK cells [33]. TMX2 has been shown to promote cytoprotective mitophagy and macroautophagy/autophagy under oxidative stress conditions [18]. Based on these findings, we hypothesized that *Tmx2* knockdown might impair embryonic development by disrupting mitophagy and overall autophagy processes. Consistent with this hypothesis, our results demonstrate that *Tmx2* knockdown inhibits both mitophagy and autophagy.

In summary, our findings indicate that *Tmx2* knockdown disrupts mitochondrial function, triggers excessive ROS production, suppresses mitophagy and autophagy, and induces apoptosis, ultimately compromising blastocyst developmental potential in mice. These results underscore the essential role of *Tmx2* in mouse preimplantation embryo development, particularly in maintaining mitochondrial function. This study elucidates the expression pattern and functional role of *Tmx2* in preimplantation mouse embryos, identifying it as a potential candidate gene associated with female infertility. Moreover, these insights contribute to a deeper understanding of the regulatory mechanisms governing preimplantation embryonic development.

## Author Contributions

**Shangrong Zhang:** conceptualization, funding acquisition, methodology, data curation, formal analysis, visualization, writing – original draft. **Qing Liu:** methodology, investigation, validation, data curation, Formal analysis. **Siyu Wang:** investigation, validation, data curation. **Xiaoyu Zhang:** investigation, validation. **Dingmei Qin:** methodology, investigation. **Ruisong Bai:** investigation, resources. **Ji Chen:** investigation, resources. **Zijian Ma:** methodology, investigation. **Zhipeng Lin:** investigation, resources. **Yuheng Bi:** investigation, resources. **Huan Liu:** investigation, resources. **Aoxue Sun:** investigation, resources. **Zhongzhi Mo:** resources. **Hongcheng Wang:** writing – review and editing. **Xiaoqing Wu:** conceptualization, funding acquisition, methodology, project administration, supervision. **Yong Liu:** conceptualization, funding acquisition, project administration, writing – review and editing.

## Disclosure

The authors have nothing to report.

## Conflicts of Interest

The authors declare no conflicts of interest.

## Supporting information


**Table S1.** KSOM Medium Composition.

**Table S2.** List of primer sets used for RT‐qPCR.

## Data Availability

The data supporting this study's findings are available on request from the corresponding author.
