# Supplementary material for: Tmx2 Maintains Mitochondrial Function to Support Preimplantation Embryogenesis
Source: FASEB J. 2025 Jun 11;39(11):e70723. doi: 10.1096/fj.202500640R (PMC12153034; doi:10.1096/fj.202500640R)
Supplement: Supplementary file 1 — Table S1. KSOM Medium Composition. [file FSB2-39-e70723-s002.docx]

***Tmx2* maintains mitochondrial function to support early embryogenesis**

The FASEB Journal

Shangrong Zhang^1^, Qing Liu^1^, Siyu Wang, Xiaoyu Zhang, Dingmei Qin, Ruisong Bai, Ji Chen, Zijian Ma, Zhipeng Lin, Yuheng Bi, Huan Liu, Aoxue Sun, Zhongzhi Mo, Hongcheng Wang, Xiaoqing Wu*, Yong Liu **

Anhui Province Key Laboratory of Embryo Development and Reproductive Regulation, Fuyang Normal University, Fuyang 236037, Anhui, China

1 These authors contributed equally to this work.

* Corresponding author: Xiaoqing Wu

ORCID: 0009-0008-9958-8655

E-mail: wuxq2018@163.com

Address: 100 Qinghe Rd, 236037, Fuyang, Anhui Province, P.R. China

**Corresponding author: Prof. Yong Liu

ORCID: 0000-0002-0583-072X

E-mail: liuyong@fynu.edu.cn

Address: 100 Qinghe Rd, 236037, Fuyang, Anhui Province, P.R. China

**Supplementary Table 1.** KSOM Medium Composition.

| Reagent Name | Mass or Volume per 250 mL |
| --- | --- |
| NaCl | 1387.5 mg |
| KCl | 46.5 mg |
| KH_2_PO_4_ | 11.9mg |
| MgSO_4_·7H_2_O | 12.325 mg |
| Glucose | 9 mg |
| Sodium Lactate | 875 μL |
| NaHCO_3_ | 525 mg |
| Sodium Pyruvate | 5.5 mg |
| CaCl_2_·2H_2_O | 62.5 mg |
| EDTA·2Na | 1.1 mg |
| L-Glutamine | 36.5mg |
| BSA | 250 mg |
| MEM Non-Essential Amino Acids Solution (100X) | 2.5 mL |
| MEM Amino Acids Solution (50X) | 5 mL |
| Antibiotic-Antimycotic (100X) | 2.5 mL |
| Water, Cell Culture Grade（Endotoxin-Free） | to 250 mL |
| Water, Cell Culture Grade（Endotoxin-Free） | to 250 mL |
