# Supplementary material for: Tmx2 Maintains Mitochondrial Function to Support Preimplantation Embryogenesis
Source: FASEB J. 2025 Jun 11;39(11):e70723. doi: 10.1096/fj.202500640R (PMC12153034; doi:10.1096/fj.202500640R)
Supplement: Supplementary file 2 — Table S2. List of primer sets used for RT‐qPCR. [file FSB2-39-e70723-s001.docx]

***Tmx2* maintains mitochondrial function to support early embryogenesis**

The FASEB Journal

Shangrong Zhang^1^, Qing Liu^1^, Siyu Wang, Xiaoyu Zhang, Dingmei Qin, Ruisong Bai, Ji Chen, Zijian Ma, Zhipeng Lin, Yuheng Bi, Huan Liu, Aoxue Sun, Zhongzhi Mo, Hongcheng Wang, Xiaoqing Wu*, Yong Liu **

Anhui Province Key Laboratory of Embryo Development and Reproductive Regulation, Fuyang Normal University, Fuyang 236037, Anhui, China

1 These authors contributed equally to this work.

* Corresponding author: Xiaoqing Wu

ORCID: 0009-0008-9958-8655

E-mail: wuxq2018@163.com

Address: 100 Qinghe Rd, 236037, Fuyang, Anhui Province, P.R. China

**Corresponding author: Prof. Yong Liu

ORCID: 0000-0002-0583-072X

E-mail: liuyong@fynu.edu.cn

Address: 100 Qinghe Rd, 236037, Fuyang, Anhui Province, P.R. China

**Supplementary Table 2.** List of primer sets used for RT-qPCR.

| Gene | Forward | Reverse |
| --- | --- | --- |
| *H2afz* | CGCAGAGGTACTTGAGTTGG | TCTTCCCGATCAGCGATTTG |
| *Tmx2* | CTGATTCTGTTCCAAGGCGG | GGTCCACAGGCTTCTCTTCT |
| *Sod1* | ATCGTGTGATCTCACTCTCAGGAG | TTTCCACCTTTGCCCAAGTCATC |
| *Sod2* | GCACCACAGCAAGCACCAC | CTGAAGAGCGACCTGAGTTGTAAC |
| *p53* | TGCTCACCCTGGCTAAAGTT | CCATGGCAGTCATCCAGTCT |
| *p21* | CCTGGTGATGTCCGACCTG | CCATGAGCGCATCGCAATC |
